# Targeting Glia with N-Acetylcysteine Modulates Brain Glutamate and Behaviors Relevant to Neurodevelopmental Disorders in C57BL/6J Mice

**DOI:** 10.3389/fnbeh.2015.00343

**Published:** 2015-12-14

**Authors:** Alice M. S. Durieux, Cathy Fernandes, Declan Murphy, Marie Anais Labouesse, Sandra Giovanoli, Urs Meyer, Qi Li, Po-Wah So, Grainne McAlonan

**Affiliations:** ^1^Department of Forensic and Neurodevelopmental Sciences, Institute of Psychiatry, Psychology and Neuroscience, King’s College LondonLondon, UK; ^2^Social, Genetic and Developmental Psychiatry Centre, Institute of Psychiatry, Psychology and Neuroscience, King’s College LondonLondon, UK; ^3^Physiology and Behaviour Laboratory, Swiss Federal Institute of TechnologySchwerzenbach, Switzerland; ^4^Institute of Pharmacology and Toxicology, University of Zurich-VetsuisseZurich, Switzerland; ^5^Department of Psychiatry, Li Ka Shing Faculty of Medicine, The University of Hong KongHong Kong, China; ^6^Department of Neuroimaging, Institute of Psychiatry, Psychology and Neuroscience, King’s College LondonLondon, UK

**Keywords:** N-acetylcysteine, magnetic resonance spectroscopy, glutamate, neurodevelopmental disorders, anxiety, prepulse inhibition

## Abstract

An imbalance between excitatory (E) glutamate and inhibitory (I) GABA transmission may underlie neurodevelopmental conditions such as autism spectrum disorder (ASD) and schizophrenia. This may be direct, through alterations in synaptic genes, but there is increasing evidence for the importance of indirect modulation of E/I balance through glial mechanisms. Here, we used C57BL/6J mice to test the hypothesis that striatal glutamate levels can be shifted by N-acetylcysteine (NAC), which acts at the cystine-glutamate antiporter of glial cells. Striatal glutamate was quantified *in vivo* using proton magnetic resonance spectroscopy. The effect of NAC on behaviors relevant to ASD was examined in a separate cohort. NAC induced a time-dependent decrease in striatal glutamate, which recapitulated findings of lower striatal glutamate reported in ASD. NAC-treated animals were significantly less active and more anxious in the open field test; and NAC-treated females had significantly impaired prepulse inhibition of startle response. This at least partly mimics greater anxiety and impaired sensorimotor gating reported in neurodevelopmental disorders. Thus glial mechanisms regulate glutamate acutely and have functional consequences even in adulthood. Glial cells may be a potential drug target for the development of new therapies for neurodevelopmental disorders across the life-span.

## Introduction

Neurodevelopmental disorders such as autism spectrum disorder (ASD) and schizophrenia affect about 1% of the population (Saha et al., 2005; Baron-Cohen et al., 2009). Their etiology is poorly understood, and treatments are limited. However, recent advances in research suggest an imbalance between excitatory (E) glutamate and inhibitory (I) GABA is a key pathophysiological feature that may explain both core and common comorbid symptoms such as anxiety (Rubenstein and Merzenich, 2003; Cortese and Phan, 2005; Lewis and Kim, 2009; Coghlan et al., 2012; Moghaddam and Javitt, 2012).

E/I imbalance in neurodevelopmental conditions can arise directly via alteration in genes encoding glutamatergic receptors or synaptic adhesion proteins (Silverman et al., 2012, 2015; Spooren et al., 2012). For example, the synapse organizers Neurexins and their binding partner Neuroligins are crucial to the formation and maintenance of excitatory and inhibitory synapses. Abnormalities in the genes encoding these proteins have been reported in both ASD and schizophrenia (Bang and Owczarek, 2013), and animal models have confirmed their role in synaptic transmission and behaviors relevant to neurodevelopmental disorders (Graf et al., 2004; Dahlhaus and El-Husseini, 2010; Grayton et al., 2013).

However, synaptic gene abnormalities account for only a relatively small proportion of the neurodevelopmental spectrum (Devlin and Scherer, 2012); and there is increasing evidence that E/I balance can also be modulated by glial mechanisms (Di Benedetto and Rupprecht, 2013). The brain’s glial support system includes astrocytes, which support and protect neurons; and microglia, the resident macrophages of the central nervous system. These cells are now appreciated to have a critical role in synapse development, maintenance and remodelling (Koyama and Ikegama), but can also influence excitatory and inhibitory synaptic transmission (Auld and Robitaille, 2003). For instance, astrocytes regulate brain levels of glutamate and GABA through the glutamate/glutamine cycle (Liang et al., 2006); and activated microglia release glutamate (Domercq et al., 2013). A role for glial cells in neurodevelopmental disorders is supported by reports of abnormalities in astrocyte gene expression in both ASD and schizophrenia (Fatemi et al., 2008; Bernstein et al., 2009); and an increased number of activated microglia in adults with ASD (Onore et al., 2012).

System x_c_−, the cysteine-glutamate antiporter found on the cell membrane of glia, is central to their influence on synaptic transmission. It can be stimulated by the compound N-Acetylcysteine (NAC, an FDA approved drug) to increase glutamate in the extrasynaptic space, thereby activating presynaptic mGluR2/3, which in turn inhibit the synaptic release of glutamate (Baker et al., 2003; Moran et al., 2005; Dean et al., 2011; Kupchik et al., 2012). Therefore, in this study we administered NAC to standard “wild-type” laboratory C57BL/6J mice to provide proof-of-concept evidence that acute modulation of glia alters glutamate levels *in vivo*; and has functional (behavioral) consequences. We used proton magnetic resonance spectroscopy (^1^H-MRS) to quantify glutamate in the left striatum, as both structural and functional abnormalities in this area are well documented in neurodevelopmental disorders (Haznedar et al., 2006; Scott-Van Zeeland et al., 2010; Simpson et al., 2010; De La Fuente-Sandoval et al., 2011; Baez-Mendoza and Schultz, 2013; Naaijen et al., 2015). In a separate cohort we assessed prepulse inhibition of startle response (PPI), a measure of sensorimotor gating which is impaired in ASD and schizophrenia (Braff et al., 2001; McAlonan et al., 2002; Kumari et al., 2003; Perry et al., 2007). We also measured anxiety in the open field arena, as this is a common comorbidity of neurodevelopmental disorders (Braga et al., 2013; Joshi et al., 2013; Matson and Cervantes, 2014).

## Materials and Methods

### Animals

Two cohorts of C57BL/6J mice (Charles River, Margate, Kent, UK) aged 7–8 weeks were used in the study. Animals were acclimatized to our facilities for a week before beginning the experimental procedures, during which they were group housed (2–4 per cage) in Tecniplast cages (32 cm × 16 cm × 14 cm) with sawdust (Litaspen premium, Datesand Ltd, Manchester), a cardboard shelter and additional bedding material (Sizzlenest, Datesand Ltd, Manchester) and maintained on a 12h/12 h light/dark cycle (07:00–19:00 h) at constant room temperature (21°C) and humidity (45%). The mice were fed a standard diet (Rat and Mouse #1 Diet, Special Diet Services, Essex, UK) and provided with water *ad libitum*. Both sexes were used in this study to avoid sex specific confounds. The oestrous phase of the female mice was not checked in this study. However, it is unlikely that this affected results because there were no major effects in the variance between males and females. All housing and experimental procedures were performed in compliance with the local ethical review panel of King’s College London, and the UK Home Office Animals Scientific Procedures Act 1986. The work was carried out under license (PPL: 70/7184) and all efforts were made to minimize animal suffering and to reduce the number of animals used.

### Drug Treatment

NAC was purchased from Sigma-Aldrich (UK), and dissolved in saline. Prior to experimental procedures, mice were injected (i.p.) with either 150 mg/kg NAC solution (30 g/L); injection volume approximately 100 microL or vehicle (saline).

### Proton Magnetic Resonance Spectroscopy of the Brain

In cohort I, 32 animals (16/sex) were treated with either NAC or vehicle, 115–175 min before data acquisition began. Animals were anaesthetized throughout the scan using an isofluorane/oxygen mix (5% induction, 2% maintenance). Body temperature and respiratory frequency were monitored and carefully regulated throughout the procedure.

**Figure 1:**
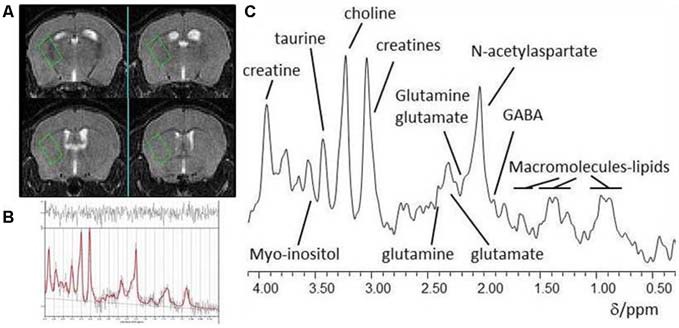
**(A)** Typical placement of a striatal voxel on T2-weighted pilot MR images of the mouse brain; **(B)** *In vivo* localized ^1^H-MR spectrum from a striatal voxel and **(C)** LCModel analysis of a ^1^H MR spectrum.

Data were acquired on a 7T horizontal bore scanner (Agilent Technologies Inc., Walnut Creek, CA, USA) using a 33 mm internal diameter quadrature volume coil (Rapid Biomedical, Rimpar, Germany). The field was shimmed to <14 MHz, width at half height of the water peak. Pilot MR images for voxel positioning were acquired using a fast spin-echo sequence with repetition *time*_(TR)_ = 1000 ms, effective echo *time*_(TE)_ = 60 ms, field of view 20 × 20 mm, 27 contiguous axial slices of 0.5 mm thickness, and two averages. These MR images were used for placement of a voxel (2.2 × 1.3 × 1.9 mm) in the left striatum for localized ^1^H-MRS as shown in Figure 1A. Point-RESolved Spectroscopy (PRESS; Bottomley, 1987) was used to acquire ^1^H-MRS data from the voxel with acquisition parameters: TR, 3000 ms; TE, 24 ms; 2048 data points; spectral width, 5208 Hz and 1000 averages. Water suppression was achieved using VAriable Pulse Power and Optimized Relaxation delays (VAPOR; Griffey and Flamig, 1990). PRESS was performed again but without water suppression with the same parameter values except collecting only eight averages from the same voxel.

Each spectrum was visually reviewed to ensure adequate signal to noise ratio, as well as the absence of artifacts. Spectra were analyzed using LCModel version 6.3–0I (Provencher, 1993) using a basis set of 21 metabolites including creatine (Cr) and glutamate (Glu). Model metabolites and concentrations used in the basis set are fully detailed in the LCModel manual (http://s-provencher.com/pages/lcm-manual.shtml). Poorly fitted metabolite peaks (Cramer-Rao minimum variance bounds of >20% as reported by LCModel) were excluded from further analysis.

### Behavioral Testing

In cohort II, 42 animals (20 males and 22 females) were treated with either NAC or vehicle 130 min prior to the assessment of open field locomotor activity. Animals were brought back to their home-cage for 10–15 min, and then PPI was performed 155 min post-treatment. Behavioral testing was carried out under stringent environmental control of housing and husbandry as well as the handling of the mice, balance and uniformity in testing, the experimenter, test location and standardized test procedures. Behavioral testing was conducted in the light phase of the light/dark cycle. After each individual test, boli and urine were removed from the test arena which was cleaned with 1% Anistel^®^ solution (high level surface disinfectant, Trisel Solution Ltd, Cambridgeshire, UK) to remove any odors. Experimenters were blind to experimental treatment group during the testing.

### Open Field Locomotor Activity

Locomotor activity in a novel open field area was assessed as a measure of anxiety as previously published (Grayton et al., 2013). Animals were placed in a square 40 × 40 cm arena under white light (8 lux) for 10 min and video recorded. The central (20 × 20 cm) and peripheral outer (remaining) areas of the arena were defined and movements in each zone were tracked with Ethovision software version 3.1 (Noldus Information Technologies bv, Wageningen, Netherlands). Time spent in the central zone was used as measure of anxiety, and distance travelled in the arena was used as measure of locomotor activity.

### Prepulse Inhibition of the Acoustic Startle Response

PPI was assessed using a chamber for mice which recorded startle response amplitude (San Diego Instruments, San Diego, CA, USA), following a previously well-validated protocol (Vuillermot et al., 2011; Labouesse et al., 2013). Animals were placed in the Plexiglas enclosure and presented with a series of discrete trials. Four different trial types were used, including pulse alone trials, prepulse alone trials, prepulse-pulse trials and no-stimulus trials (where no sound was played other than the constant background noise). The pulse and prepulse stimuli consisted of a sudden elevation of the broadband white noise from the background level of 65 dB_A_ with a rise time of 0.2–1 ms, for 40 and 20 ms respectively. Three pulse intensities (100, 110 and 120 dB_A_), and three prepulse intensities (71, 77 and 83 dB_A_—which corresponded to 6, 12 and 18 dB_A_ above the background noise respectively) were used. The onset of the pulse was presented 100 ms after the onset of the prepulse, for all prepulse-pulse trials.

Animals were allowed to adapt to the enclosure for 2 min before the beginning of the first trial. They were presented with six pulse only trials (two trials of each intensity) for habituation, which were not included in the analysis. The test phase consisted of 10 blocks of 16 discrete trials. Each block included three pulse alone trials (100, 110 and 120 dB_A_), three prepulse alone trials (71, 77 and 83 dB_A_), nine prepulse-pulse trials (all possible prepulse-pulse combinations), and one no-stimulus trial. Within each block, the 16 trials were presented in pseudorandom order and separated by a variable interval of 10–20 s (average 15 s).

For each trial type, average reactivity was calculated over the whole experiment, excluding the first six pulse alone trials. PPI was measured as the percent inhibition of startle response (%PPI) in pulse only trials. For each animal, it was calculated as follows: [(mean reactivity on pulse alone trials) − (mean reactivity on prepulse-pulse trials)/(mean reactivity on pulse alone trials)] * 100; for each pulse intensity (100, 110 and 120 dB_A_), and each prepulse intensity (71, 77 and 83 dB_A_).

## Statistical Analysis

^1^H-MRS and open field data were analyzed using a general linear model (IBM SPSS Statistics version 21) including group and sex as between subject factors. The time post-dose, t_post-dose_ was included as covariate in the analysis of metabolite concentrations, and correlations between metabolite concentrations and t_post-dose_ were explored using Pearson correlation coefficients.

For PPI analysis, reactivity to pulse alone trials were analyzed in a 3 × 2 × 2 repeated measures ANOVA (pulse level × group × sex). %PPI data were analyzed in 3 × 3 × 2 × 2 repeated measure ANOVA (prepulse level × pulse level × group × sex). Where there was a significant interaction between sex and another factor on %PPI, data were re-analyzed separately for each sex.

## Results

### Localized ^1^H-MRS of the Brain

#### Creatine (Cr)

A representative ^**1**^H-MRS spectrum of the left striatum is shown in Figures 1B,C. There was no main effect of treatment or sex on creatine (from creatine and phosphocreatine; all *F*_(1,26)_ < 0.3, all *p* > 0.6). All subsequent metabolite concentrations were therefore calculated in reference to Cr to account for potential inter-individual differences in voxel composition (Gussew et al., 2012).

#### Glutamate (Glu)

**Figure 2:**
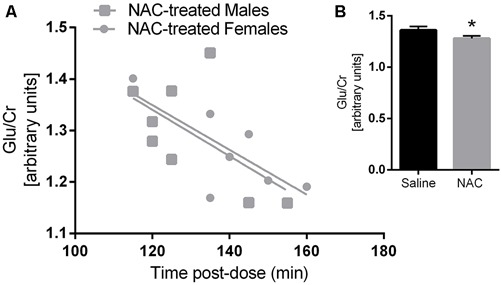
**Glutamate/Creatine was negatively correlated with time post-dose in the N-acetylcysteine (NAC)-treated group (A) but not in the saline group.** Glutamate/Creatine levels were significantly lower in NAC treated animals **(B)** *p* < 0.05.

Glu/Cr concentrations were significantly lower in the NAC treated group, as indicated by a main effect of treatment (*F*_(1,26)_ = 5.77, *p* = 0.02). There was also a main effect of t_post-dose_ on Glu/Cr (*F*_(1,26)_ = 10.2, *p* = 0.004). Furthermore Glu/Cr was negatively correlated with t_post-dose_ in the NAC group (*r* = −0.657, *p* = 0.008), but not in the vehicle group (*r* = −0.417, *p* = 0.108); as there was no main effect of sex on Glu/Cr, both sexes were examined together for the correlation analysis. Please refer to Figure 2.

### Open Field Locomotor Activity

#### Distance Travelled

**Figure 3:**
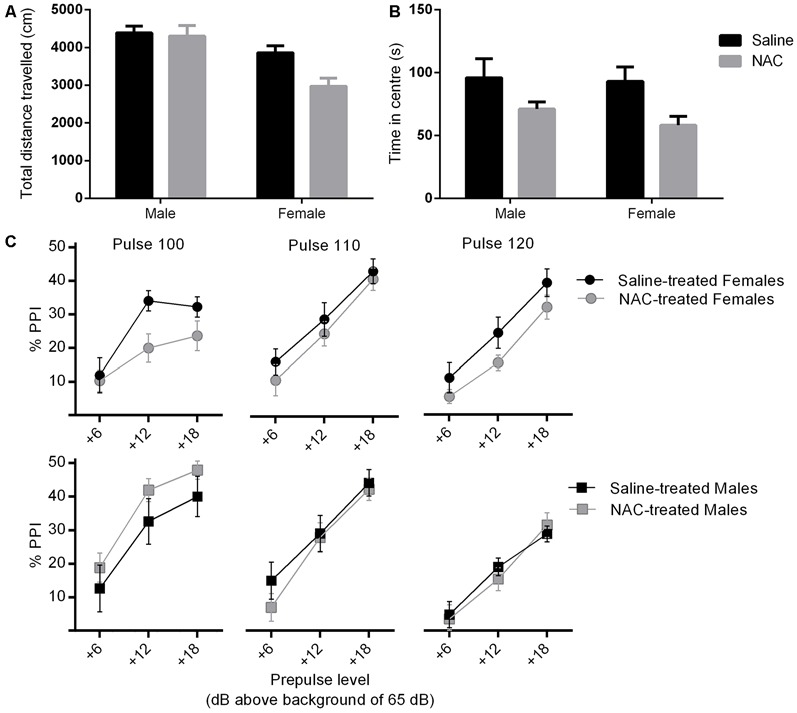
**NAC treatment reduced locomotor activity (main effect of treatment, *p* = 0.033) (A) and increased anxiety (main effect of treatment, *p* = 0.007) (B), in females more than males.** In **(C)**, percent prepulse inhibition (%PPI) is shown as a function of the three pulse levels (100, 110 or 120 dB_A_), and the three prepulse levels (6, 12 or 18 dB_A_ above background level of 65 dB_A_). %PPI was reduced by NAC treatment in females only (main effect of treatment, *p* = 0.032).

There were main effects of treatment (*F*_(1,38)_ = 4.90, *p* = 0.033) and sex (*F*_(1,38)_ = 17.98, *p* < 0.001) on distance travelled in the arena of the open field, and no sex × treatment interaction. NAC treated mice travelled significantly less than their saline treated counterparts. Overall females travelled less distance than males (please refer to Figure 3A).

#### Time in Central Zone

As shown in Figure 3B, there was a main effect of treatment on time spent in central zone (*F*_(1,38)_ = 8.22, *p* = 0.007), but no main effect of sex and no sex × treatment interaction. NAC treated animals spent significantly less time in the central zone. This treatment main effect remained significant when co-varying for distance travelled (*F*_(1,37)_ = 8.39, *p* = 0.006), which indicates that the difference was not likely to be due to a change in locomotor activity.

### Prepulse Inhibition (PPI) of the Acoustic Startle Response

Mean reactivity was higher at higher pulse intensity in pulse alone trials, as indicated by a mains effect of pulse intensity (*F*_(1.8, 67.7)_ = 689.4, *p* < 0.001). There was also a main effect of sex on reactivity to pulse alone trials (*F*_(1,38)_ = 66.23, *p* < 0.001), as well as a pulse × sex interaction (*F*_(1.8, 67.7)_ = 41.4, *p* < 0.001).

There was no main effect of treatment or sex on %PPI. The treatment × sex interaction approached significance (*F*_(1,38)_ = 3.11, *p* = 0.086), therefore the analysis was repeated in each sex separately (please refer to Figure 3C). There was no effect of treatment on %PPI in males. However NAC treated females displayed a significant reduction in %PPI compared with controls (*F*_(1,20)_ = 5.29, *p* = 0.032). The disruption of PPI by NAC emerged independently of specific pulse and prepulse intensities, as shown by non-significant interactions of pulse and prepulse with treatment.

## Discussion

This study provided proof-of-concept evidence that glutamate levels and behavior can be altered acutely by extra-synaptic mechanisms targeting glial cells. Activation of system x_c_− by NAC lowered glutamate concentrations in the striatum in a time-dependent fashion. NAC-treated animals displayed reduced locomotor activity in the open field as well as increased anxiety, which was independent from general activity levels. NAC treatment reduced the prepulse inhibition of startle response in females exclusively.

The acute glutamatergic effects of NAC observed in this study are consistent with, and extend previous findings. For example previous studies showed that NAC reduces excitatory currents in an mGluR2/3 dependent fashion (Moran et al., 2005; Kupchik et al., 2012). One ^1^H-MRS study in a mouse model of schizophrenia found that chronic NAC treatment reduces cortical glutamate levels in developing animals (Das Neves Duarte et al., 2012). Here, we show that one dose of NAC is sufficient to lower striatal glutamate in a time-dependent fashion; the lowest glutamate levels are evident 120 min post-dose and continue to decrease for at least 60 min. We report that a single dose of NAC also disrupts behavior from 120 min post-administration. We interpret the behavioral differences as likely due to a glutamate decrease, but acknowledge the evidence is indirect. These findings may have translational importance as they suggest that targeting glia can shift glutamate levels; and this is relevant to neurodevelopmental disorders such as ASD and schizophrenia, where glial abnormalities and E/I imbalance are prominent features.

Our finding of reduced striatal glutamate is analogous to the decrease of glutamate and its metabolite glutamine observed in the striatum of adult males with ASD (Horder et al., 2013). Our observations of greater anxiety and impaired sensorimotor gating in NAC-treated animals also mimics impairments commonly found in ASD and related neurodevelopmental disorders (McAlonan et al., 2002; Perry et al., 2007; Braga et al., 2013; Joshi et al., 2013; Matson and Cervantes, 2014). Thus, although our design did not make it possible to assess glutamate levels and behavior in the same animals, behavioral findings may well relate to a change in glutamate concentrations.

For example, locomotor activity, anxiety and PPI at least partly depend on glutamatergic transmission (Takeuchi et al., 2001; O′Neill et al., 2003; Wieronska and Pilc, 2013; Saitoh et al., 2014). Here, we report that indirect mGluR2/3 agonism via NAC causes lower activity. This is consistent with a role for mGluR2/3 in the tonic inhibition of locomotion; and the observation that mGluR2/3 antagonism increases locomotion (O′Neill et al., 2003). However, mGluR2/3 agonism has been reported to both increase (Imre et al., 2006; Satow et al., 2008) and decrease (Imre et al., 2006; Grivas et al., 2013) anxiety, depending on the dose. Similarly, acute administration of mGluR2/3 agonists has been reported to have either no effect (intra-peritoneal administration of LY379268; Hikichi et al., 2010), or impair (intracerebral administration of *L*-CCG-I; Grauer and Marquis, 1999), PPI. Thus, the mGluR2/3 response is likely to be complex and depend on the dose and administration regime of compounds targeting these receptors.

Although we show here that NAC administration mimics lower striatal glutamate and causes behavioral abnormalities similar to those found in ASD, paradoxically, NAC has shown clinical benefits in small scale double blind trials in this condition (Hardan et al., 2012; Ghanizadeh and Moghimi-Sarani, 2013). However, the latter study examined NAC as an adjunct alongside risperidone, and a recently completed Clinical Trial of NAC in ASD did not find evidence of efficacy (K. Gray, personal communication). There are a number of possible explanations for these inconsistencies. First, the acute effects of NAC may be quite distinct from the effects of repeated administration in clinical settings. Arguably, the glutamate system responds differently to chronic modulation compared to acute challenge. Secondly, the developmental stage studied may be critical, as glutamate levels in humans are known to vary with age (Segovia et al., 2001; Kaiser et al., 2005). The clinical trials of NAC in ASD were mostly completed in children (Hardan et al., 2012; Ghanizadeh and Moghimi-Sarani, 2013), while we used adult animals. Third, the effects of NAC may be different in a “disordered” system. We used standard in-bred laboratory mice in this study; it is possible that the effects of NAC would be different in a mouse model showing baseline E/I imbalance. Some support for this latter suggestion comes from preliminary work in schizophrenia. NAC has shown benefits in adults with schizophrenia (Zavodnick and Ali, 2014), but a study in healthy volunteers found that NAC pretreatment worsened auditory mismatch negativity following ketamine administration (Gunduz-Bruce et al., 2012), a paradigm thought to mimic psychosis. Finally, it is possible that the clinical benefits of chronic administration of NAC are in fact mediated by its antioxidant actions, rather than its glutamatergic action (Dean et al., 2009; Rushworth and Megson, 2013). Thus, we emphasize that our findings do not speak to the therapeutic use of NAC in neurodevelopmental disorders, for which there is no clear consensus. Rather, we suggest that our finding shows that modulating glutamate and behavior via glial mechanisms is possible, and suggests that glial mechanisms may be a tractable target for drugs which aim to modulate E/I.

We acknowledge some unexpected findings in this study. We elected to study NAC in both female and male animals and found sex-differences in the PPI response following NAC administration. PPI is particularly sensitive to sex-differences in both laboratory animals (Lehmann et al., 1999; Ralph et al., 2001; Zhang et al., 2015) and individuals with neurodevelopmental disorders (Kumari et al., 2004; Gogos et al., 2009). Moreover, although there were no treatment × sex interactions in measures of activity and anxiety, the effects of NAC on these measures was numerically greater in females compared to males. It is therefore possible that female mice are more sensitive to the behavioral consequences of the change in glutamate, however larger sample sizes would be needed to explore this in more detail. It would also be valuable to determine whether there are any sex differences in glial mechanisms. We echo the call for more research into sex differences in neurodevelopmental disorders, particularly as it becomes apparent that their prevalence in females may be higher than thought previously (Brix et al., 2015). We also acknowledge that an important limitation of this study was that we did not measure glutamate levels and assess behavior in the same animals. The current design was chosen to avoid confounds related to putting the same animals through several experimental procedures sequentially. Previous work has shown that invasive behavioral testing could induce changes in neural activity patterns (Xu et al., 2012), and brain metabolite levels (Zhou et al., 2012). On the other hand, the imaging procedure, which required 2 h of anaesthesia, is a significant exposure and could impact upon subsequent behavioral testing. Therefore we chose to use a naïve cohort of mice animals for the behavioral and the imaging experiments. Future research will aim to correlate E/I and behavioral measures in the same animals, possibly using* ex vivo* methods. Finally, the PRESS spectroscopy acquisition protocol we used did not allow for estimation of GABA concentration. Glutamate and GABA are constantly in flux and measuring the other side of the E/I ratio would be useful to obtain a fuller picture in the future.

In summary, this work confirms that glial mechanisms regulate glutamate acutely and have functional consequences in adult animals. Glial cells may therefore be a potential drug target for the development of new therapies for neurodevelopmental disorders even in the adult system.

## Author Contributions

AMSD, GM, P-WS, CF, DM, QL, ML, SG and UM designed the research. AMSD, P-WS, CF, ML, SG and UM acquired the data. AMSD, GM, P-WS, CF, ML, QL, SG and UM analyzed the data. AMSD, GM, P-WS, CF, DM, ML, QL, SG and UM critically interpreted the data. AMSD and GM drafted the manuscript. AMSD, GM, P-WS, CF, DM, ML, QL, SG and UM approved the final version.

## Funding

AMSD is supported by a PhD studentship from the National Institute for Health Research (NIHR) Biomedical Research Center at South London and Maudsley NHS Foundation Trust and King’s College London. The views expressed are those of the author(s) and not necessarily those of the NHS, the NIHR or the Department of Health. This work was funded by the Sackler Institute for Translational Development.

## Conflict of Interest Statement

The authors declare that the research was conducted in the absence of any commercial or financial relationships that could be construed as a potential conflict of interest.
